# Oral Hygiene Recommendations From Orthodontists to Orthodontic Patients

**DOI:** 10.1111/idh.70028

**Published:** 2026-02-11

**Authors:** Christina Baumer, Irene Schmidtmann, Daniela Ohlendorf, Priscila Ferrari Peron, Ambili R. Mundethu, Heinrich Wehrbein, Christina Erbe

**Affiliations:** ^1^ Department of Orthodontics University Medical Center of the Johannes Gutenberg‐University Mainz Germany; ^2^ Institute for Medical Biostatistics, Epidemiology and Informatics (IMBEI) University Medical Center of the Johannes Gutenberg‐University Mainz Germany; ^3^ Institute of Occupational Medicine, Social Medicine and Environmental Medicine Goethe‐University Frankfurt am Main Germany

**Keywords:** demineralisation, oral hygiene, orthodontics, tooth brushing techniques

## Abstract

**Intro:**

The aim of this questionnaire survey was to analyse the approach orthodontists take on oral hygiene (OH) throughout Germany. The information orthodontists provide their patients with respect to brushing and steps the patients can take at home to achieve the best results, as well as the amount of times orthodontists scheduled controls and appointments with their patients was collected.

**Methods:**

A computer program randomly selected 1000 members from the DGKFO directory to whom questionnaires were mailed. The size and population of the federal states (FS) were taken into account. The FS with the highest return rates (Baden‐Württemberg (BW), Bavaria (B), Hesse (H), Lower Saxony (NDS), North Rhine‐Westphalia (NRW)) were considered and differences between the 16 FS were divided into north, south, east and central. Data analysis was performed using cross tables and chi‐square tests.

**Results:**

The response rate of the questionnaires was 52.4%. The majority (53.8%) worked in one practice alone. Most (59.1%) have been orthodontists for 5–25 years. OH instructions were given orally by the dental assistant (> 90%). Multibracket appliances (MBA) were checked monthly, and removable appliances (RA) every 2 months; in over 90% at each session. Professional dental cleaning (PDC) took place every 6 months, except in NDS (every 3 months). When considering tooth brushing methods, the bass technique was specified for MBA and the Fones‐technique for RA.

**Conclusion:**

The dental assistant gave the subjects instructions regarding oral care at home. The MBA control was carried out monthly and at RA every 2 months. PDC usually took place every 6 months.

## 1 Introduction

Demineralisation is one of the most common undesirable side effects of multibracket treatment [1, 2, 3]. Young patients with inadequate oral hygiene at the beginning of treatment have a higher risk of demineralisation with fixed appliance therapy [4, 5, 6, 7]. Plaque index and gingival index may increase or an unpleasant halitosis may occur as early as 1 week after integration [8]. The development of white spot lesions (WSL) is directly related to plaque accumulation around the brackets [9, 10]. These create additional retention surfaces that are difficult to clean for the patient [11, 12]. More plaque also implies more caries‐inducing bacteria [13, 14, 15]. An increase of *Streptococcus mutans* in saliva and plaque can be detected throughout the entire orthodontic treatment, thus increasing the odds of developing gingivitis and can subsequently also lead to periodontal disease [16, 17]. WSL can become visible just 4 weeks after the start of the treatment [1]. This is why optimal brushing technique, detailed controls as well as oral hygiene instruction and regular professional dental cleanings are so important. Several clinical studies describe the positive effects of oral hygiene programs [18, 19, 20] and individual oral hygiene instructions during orthodontic treatments [21, 22, 23, 24, 25, 26]. The repeated motivation and oral hygiene instructions every 3 weeks lead to plaque reduction and to a better understanding of the correct tooth brushing technique [27].

Yeung et al. [20] discussed the effectiveness of an oral hygiene program for adult patients during orthodontic treatment. The test group went through an intensive oral hygiene program while the control group did not, and continued with their usual oral hygiene routine. Papillary Bleeding Index (PBI) and Gingival Index (GI) were statistically significantly lower in the test group (PBI: 1.39 ± 0.25, *p* < 0.001; GI: 3.94 ± 0.48, *p* < 0.032) than in the control group (PBI: 3.24 ± 0.43, *p* = 0.021; GI: 5.45 ± 0.43, *p* = 0.001). The plaque index (PI) of the test group was slightly higher (*p* < 0.07) than that of the control group. However, an overall improvement of PI, GI and PBI was observed in both groups. Yeung et al. concluded that oral hygiene programs have a positive effect on oral health. Another study conducted in 2013 with 42 children with MBA showed that a short weekly text message reminding the parents of the importance of brushing teeth ad a positive effect on PI, GI and PBI [28].

Since orthodontic treatment can take several months to years, it is important to give the patient an understanding of all options for optimal dental care before and during treatment [29, 30] and to provide repeated motivation and regular PDC throughout the treatment [31, 32].

Most of the population uses their own personal brushing technique, which usually comes closest to the horizontal scrubbing [9, 33, 34, 35] method [36, 37]. Dental associations, industries, books and publications most often refer to the Bass technique [33] and the Modified Bass technique [36, 38].

In 2003, in a study by Poyato‐Ferrera et al. [36], patients used their usual tooth brushing technique and afterwards the trained Modified Bass technique. The supragingival plaque was significantly lower compared to the usual cleaning technique. In 2012, in a computer‐assisted study, Harnacke et al. [39] compared the Fones rotation technique [33] with the Modified Bass technique and a control group No significant differences could be found between the Bass group and the control group. The Fones technique proved to be superior in terms of both learning the technique and the measurement values such as the PBI. These differences were statistically significant (*p* = 0.035 for PBI after 28 weeks).

Therefore, the aim of this questionnaire—based study was to assess oral hygiene recommendations with regard to optimal oral hygiene, regular instructions and the tooth brushing method used. Above all, it is important for our daily work routine as orthodontists as well as for dental hygienists to be informed about the most common oral hygiene recommendations given to our patients.

## 2 Methods

### 2.1 Study Population and Methodology

This cross‐sectional questionnaire study was designed in cooperation with the Institute of Medical Biometry, Epidemiology and Computer Science (IMBEI). From the directory of the German Society for Orthodontics (DGKFO) 1000 orthodontists across the 16 federal states (FS) of Germany were randomly selected using a computer generated randomisation list and sent a questionnaire by post. Stratified sampling was used and the size and number of inhabitants of each respective federal state was taken into consideration. The questionnaire consisted of 22 questions about the orthodontists and their favoured oral hygiene instructions, how they controlled their patient's oral care and a feedback section. The orthodontists were informed about the anonymity of their answers and that the IMBEI was involved in the handling of the data. The questionnaires were stored in locked cabinets only the principal investigator and data manager had access to. They were also the only one with access to the computers used. All study staff involved in the study are subject to a confidentiality agreement.

Questionnaires were sent out in January 2012 and arrived back in Mainz in May 2012 at the latest. Only submitted questionnaires that were completely filled out were evaluated.

The questionnaires were pre‐tested by eight postgraduate students who filled out the questionnaire and gave feedback.

#### 2.1.1 Demographic Classification

The location of each practice was organised based on the respective postal code of the federal state that it was in. The federal states with the highest return rate (Baden‐Württemberg, Bavaria, Hesse, Lower Saxony, North Rhine‐Westphalia) were examined. The differences between the 16 federal states were divided into Northern, Southern, Eastern and Central Germany (Table 1) on the one hand and into Western and Eastern Germany (Table 2) on the other hand. These demographic classifications were made according to the Federal Institute for Research on Building, Urban Affairs and Spatial Development (BBSR, Berlin Germany).

**TABLE 1:** Demographic classification of the German federal states in north, central, south and east according to BBSR.

North	Bremen
Central	Hamburg
	Lower Saxony
	Schleswig‐Holstein
	Hesse
	North Rhine‐Westphalia
	Saarland
South	Baden‐Wuerttemberg
	Bavaria
	Rhineland‐Palatine
East	Berlin
	Brandenburg
	Mecklenburg‐West‐Pomerania
	Saxony
	Saxony‐Anhalt
	Thuringia

**TABLE 2:** Demographic classification of the German federal states in East and West Germany according to BBSR.

Ost
Brandenburg
Mecklenburg‐West‐Pomerania
Saxony
Saxony‐Anhalt
Thuringia
Former East Berlin
West
Bremen
Hamburg
Lower Saxony
Schleswig‐Holstein
Hesse
North Rhine‐Westphalia
Saarland
Baden‐Wuerttemberg
Bavaria
Rhineland‐Palatine
Former West Berlin

#### 2.1.2 Data Analysis

The data were evaluated using the SPSS Statistics 22 statistics program (IBM, Armonk, USA) and/or the Excel 2010 program (Microsoft, Redmond, USA). Absolute and relative frequencies were presented in cross tables and compared between the individual FS as well as the northern, eastern, southern and central parts of Germany using a chi‐square test.

As this is an explorative study, *p*‐values were not adjusted with the repetition of tests and should therefore be only viewed as descriptive data.

## 3 Results

The response rate of the questionnaires was 52.4%. Of 524 returned questionnaires, 128 (24.4%) came from North Rhine‐Westphalia, 90 (17.2%) from Bavaria, 87 (16.6%) from Baden‐Württemberg, 51 (9.7%) from Hesse and 45 (8.6%) from Lower Saxony. Based on the demographic division into north, central, south and east, 201 (38.4%) of the questionnaires came from the south. From central Germany came 186 (35.5%) questionnaires, 69 (13.2%) came from the east and 68 (13.0%) came from the north. The contrast between the questionnaires returning from west and east Germany was large, as 59 (11.3%) questionnaires were received from the east and 465 (88.7%) from the west. The majority (353) of the 524 orthodontists were between the ages of 36 and 55 years (67.3%). A quarter (131) of respondents were older than 55 years and 40 (7.6%) were younger than 36 years. The majority (380) of the 524 (72.5%) orthodontists had been working in this profession for more than 10 years. Among these orthodontists, 162 (30.9%) had 11–15 years of work experience and 177 (33.8%) had been working for more than 25 years. A total of 144 of the 524 (27.5%) respondents had up to 10 years of orthodontic work experience and 107 (20.4%) had been working for 5–10 years. Only 37 orthodontists (7.1%) had been working for < 5 years. In all of Germany (512 of the 524 orthodontists), as well as in the individual 5 federal states with the most returned questionnaires, the respondents stated that the dental assistants (DA) carried out the oral hygiene instructions in over 90% of the cases. The oral explanation was with 95.2% (499 of the 524 votes) most frequently indicated, followed by 399 (76.1%), who stated the execution of cleaning training and 270 (51.5%) the use of an informational brochure. Almost all [496 (94.7%)] of the 524 surveyed orthodontists, stated that they personally assess the oral hygiene of their patients during each visit. The majority (391) of the 524 (74.6%) orthodontists surveyed stated that they schedule monthly check‐ups for patients with fixed appliances. In comparison, 367 (70%) of orthodontists recommended that patients with RA have an oral hygiene check up every 2 months.

**FIGURE 1:**
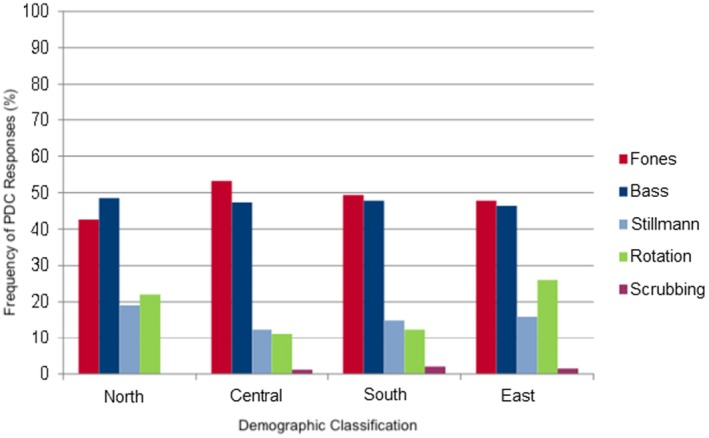
Bar chart describing the frequency of responses (%) to perform a PDC in relation to all of Germany.

The data on the frequency of PDC varied (Figure 1 and Table 3): The orthodontists in central (40 of the 128 (31.3%) interviewed), southern (58 of the 141 (41.1%) orthodontists) and eastern Germany (19 of the 48 (39.6%) interviewed) recommended a PDC ‘every 6 months’. In the north, 13 of the 32 (40.6%) respondents recommended the PDC ‘every 3 months’. The values collected from all of Germany were in line with the results of each of the five individual federal states. In four of the five federal states (BW, B, H, NRW) the orthodontists recommended a PDC ‘every 6 months’. ‘Every 3 months’ was recommended less frequently. Lower Saxony (northern Germany) was the only federal state (out of the five), where 8 of the 20 (40%) orthodontists recommended a PDC ‘every 3 months’, four out of 20 (20%) preferred ‘every 6 months’ and only some recommended every 2 or 12 months. On average, however, < 10% of respondents gave these answers.

**TABLE 3:** Frequency of responses regarding the performance of a PDC in percent in relation to the whole of Germany.

Regions	Every 2 months (%)	Every 3 months (%)	Every 4 months (%)	Every 6 months (%)	Every 12 months (%)
North	6.5	40.6	9.5	15.6	6.5
Central	10.5	25	8.5	31.3	9.5
South	6.5	26.2	10.5	41.1	7
East	6	35.4	4	39.6	8.5

### 3.1 Cleaning Technique for Removable Appliances

#### 3.1.1 Fones Technique

**FIGURE 2:**
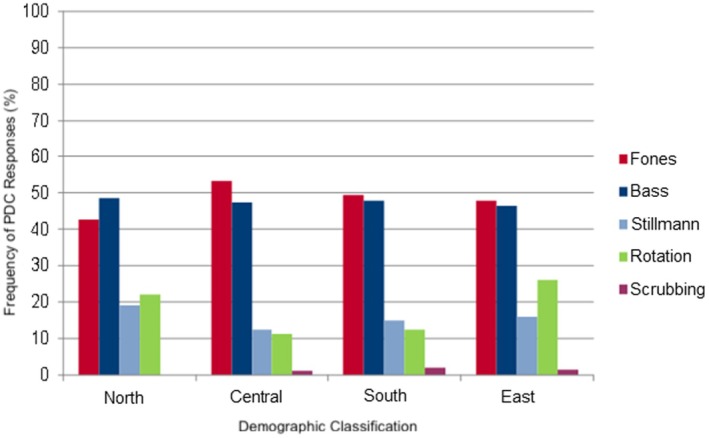
Bar chart describing the frequency of responses (%) for tooth brushing techniques with removable appliances, in relation to all of Germany.

The Fones technique was recommended by 29 of the 68 (42.6%) orthodontists in northern Germany, 99 of the 201 (49.3%) in southern Germany and 33 of the 69 (47.8%) orthodontists in eastern Germany. In central Germany, 99 of the 186 (53.2%) orthodontists indicated the Fones technique (Figure 2 and Table 4). When considering the data collected from the five federal states that had the most returned questionnaires, 42.5% of orthodontists from Baden‐Württemberg and 55.6% of Bavarian orthodontists recommended the Fones technique.

**TABLE 4:** Frequency of responses regarding removable appliances in percent in relation to the whole of Germany.

Regions	Fones (%)	Bass (%)	Stillmann (%)	Rotation (%)	Scrubbing (%)
North	42.6	48.5	19	22	0
Central	53.2	47.3	12.5	11.3	1.3
South	49.3	47.8	15	12.4	2
East	47.8	46.4	16	26	1.5

#### 3.1.2 Bass Technique

An average of 47.5% of orthodontists in northern, central, southern and eastern Germany combined, recommended the Bass technique (N: 48.5%, C: 47.3%, S: 47.8%, E: 46.4%) (Figure 2 and Table 4). Over 40% of orthodontists in Bavaria, Lower Saxony and North Rhine‐Westphalia recommended Bass technology. In Baden‐Württemberg, 45 of the 87 (51.7%) orthodontists also preferred this cleaning technique as well as 28 of the 51 (54.9%) orthodontists in Hesse.

#### 3.1.3 Stillman Technique

The Stillman Technique had values between 10% and 20% throughout Germany, as well as in the five individual FS (Figure 2 and Table 4).

#### 3.1.4 Rotation Technique

The rotating method reached a value of over 20% in the north as well as in the east. This technique was preferred by just over 10% of orthodontists in central and southern Germany (C: 11.3%, S: 12.4%) (Figure 2 and Table 4). Of the five FS with the most returns, Lower Saxony had a 20% recommendation rate for this method. The remaining four federal states recommended the circular technique with an average of 10.5% (H: 5.9%—BW: 13.8%).

#### 3.1.5 Horizontal Scrubbing Technique

The horizontal scrubbing technique was reported throughout Germany and in the five separately considered FS with values between 0% and 3.4% (Figure 2 and Table 4).

### 3.2 Cleaning Technique for Fixed Appliances

#### 3.2.1 Fones Technique

**FIGURE 3:**
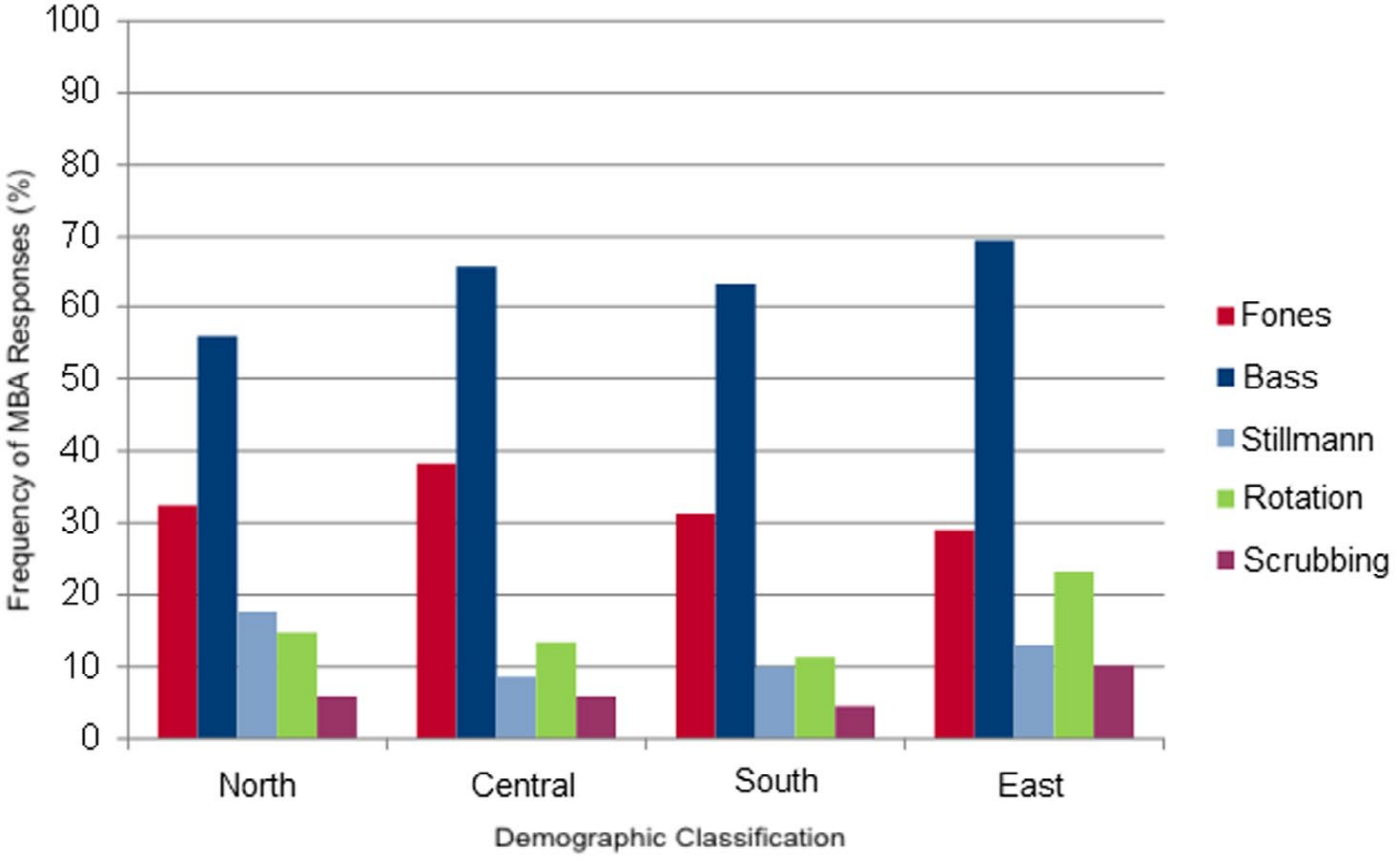
Bar chart describing the frequency of responses (%) for tooth brushing techniques in multibracket appliances, in relation to all of Germany.

The Fones technique was popular among 29.0% of orthodontists in eastern Germany and 38.2% in central Germany (Figure 3 and Table 5). All five federal states together had an average recommendation of 33.5% (between 27.6% in BW and 38.3% in NRW).

**TABLE 5:** Frequency of responses regarding MBA in percent in relation to the whole of Germany.

Regions	Fones (%)	Bass (%)	Stillmann (%)	Rotation (%)	Scrubbing (%)
North	32.5	55.9	17.6	14.7	5.9
Central	38.2	65.6	8.6	13.4	5.9
South	31	63.2	10	11.4	4.5
East	29.0	69.6	10.3	23.2	10.1

#### 3.2.2 Bass Technique

The Bass technique was concluded to be the cleaning technique of choice for fixed MBA. In northern Germany, 38 of the 68 orthodontists (55.9%) voted for this technique; in central Germany 122 of the 186 respondents (65.6%); in southern Germany 127 of the 201 orthodontists (63.2%); and in eastern Germany 48 of the 69 (69.6%) orthodontists (Figure 3 and Table 5). Baden‐Württemberg [60 of 87 (69.0%) orthodontists], Bavaria [54 of 90 (60.0%) orthodontists], Hesse [36 of 51 (70.6%) orthodontists] and North Rhine‐Westphalia [85 of 128 (66.4%) orthodontists] clearly recommended the Bass technique. In Lower Saxony, the recommendation was less clear at 48.9% (22 of the 45 orthodontists).

#### 3.2.3 Stillman Technique

The Stillman technique had a recommendation value of just over 20% in Lower Saxony [11 of the 49 (24.4%) orthodontists] (Figure 3 and Table 5).

The rest of Germany (between 8.6% in central Germany and 17.6% in the north), as well as the five FS (between 7.8% in NRW and 24.4% in NDS) rarely recommend this technique.

#### 3.2.4 Rotation Technique

This method was not recommended by orthodontists in northern [58 of 68 (85.3%) respondents], central [161 of 186 (86.6%) respondents], southern [178 of 201 (88.6%) respondents] and eastern Germany [53 of 69 (76.8%) respondents] (Figure 3 and Table 5). The orthodontists of the five FS did not recommend this technique either.

#### 3.2.5 Horizontal Scrubbing Technique

This technique was recommended by 4.5% of orthodontists from the south and 5.9% in central and northern Germany. Seven of the 69 (10.1%) orthodontists in the east, and very few orthodontists in the five FS recommended this method (Figure 3 and Table 5) (between 2.2% in Lower Saxony and 6.3% in North Rhine‐Westphalia).

A comparison of the two diagrams (Figure 2, Table 4 and Figure 3, Table 5) shows that Bass and Fones techniques are by far the most popular brushing techniques. In the case of RA, both the Fones and Bass techniques were mentioned. The Bass technique was recommended for multibracket appliances. Stillman and scrubbing techniques were regarded by only a few orthodontists as the tooth brushing technique of choice for both appliances. However, the scrubbing technique achieved higher values with MBA than with RA.

## 4 Discussion

Various tooth brushing techniques are described in literature as the most preferred method [34, 36, 37, 38, 39, 40, 41, 42, 43]. The regular performance of professional dental cleanings [32, 44, 45, 46, 47] and the necessity of oral hygiene instructions [18, 19, 21, 22, 23, 24, 27] are undisputed. However, as there are no clear indications as to what exactly the majority of German orthodontists actually recommend, a questionnaire with the above topics was sent randomly to 1000 orthodontists from the DGKFO list as part of this study. The response rate was 52.4%.

In 2008, Eichenauer et al. [48] achieved a response rate of 99.5% in their telephone survey dealing with the cleaning of removable orthodontic appliances. This suggests that a personal interview is more likely to encourage respondents to participate in such a study than an impersonal questionnaire that requires additional work to complete and return. However, a telephone survey with a number of 20 questions, some of which had several possible answers, would have been very difficult. This would have taken a lot of time and might have resulted in other orthodontists not participating. With this in mind, the questionnaire study, which was sent by post and could be completed manually at any time, was the right decision.

An online survey Rosenstiel et al. [49] published in 2004 shows that sending the questionnaire by email does not improve the amount of responses. Suzuki et al. [50] achieved significantly better results with their online survey in 2015 than Rosenstiel with a response rate of 52.46%. Many other scientists also used a questionnaire to obtain information [49, 50, 51, 52, 53].

The questionnaire is also an effective method in reaching as many orthodontists as possible, and being able to pose questions on a broad spectrum of topics.

### 4.1 Oral Hygiene Instructions and Control

In the present study, 95.2% of the orthodontists questioned verbally share instructions on oral hygiene with their patients. Cleaning training is recommended by 76.1% of orthodontists. Only 51.5% of orthodontists reported using an informational brochure. In over 90% of cases, oral hygiene instructions are carried out by the DA. This is likely credited to the high level of training that DAs receive nowadays. Specially trained dental hygienists train and instruct patients how to properly take care of their oral health, and thus are responsible for the prevention of caries, WSL and gingivitis. Dental hygienists also participate in their diagnosis and treatment [54, 55]. Depending on the country, these dental care professionals administer local anaesthesia, apply sealants and even place caries restorations [56]. For this reason it is essential to point out their important role in the provision of oral hygiene instructions. The tendency towards oral education and cleaning training corresponds to the information in the literature [18, 19, 20] as well as the positive effect that individual instructions have on a patient's oral hygiene [21, 22, 23, 24, 25, 26, 28]. In their study, Harnacke et al. [39, 41] examined the differences between different distribution methods of information: orally standardised, written and orally individualised. The ‘orally individualised’ method achieved significantly better results in terms of PBI and oral hygiene skills. In another study, Harnacke et al. [39] also proved that individualised oral hygiene instructions are the best motivation for patients. In contrast, Lim et al. [57] could not find any differences between personal instructions, written instructions and video instructions. The orthodontists interviewed in this study stated that they schedule monthly check‐ups for patients with fixed appliances and every 2 months for patients with RA. This could be due to the fact that fixed appliances can only be cleaned in the mouth, and that the undercut areas prove to be challenging to clean—making the entire cleaning process more difficult than that for RA. Repeated motivation and instruction in patients with a fixed MBA approximately every three to 4 weeks has a positive effect on their oral health [27, 58].

### 4.2 Performance of a Professional Dental Cleaning

Central, southern and eastern Germany stated that a PDC should be carried out ‘every 6 months’. Orthodontists in northern Germany (40.6%) recommended a PDC ‘every 3 months’. Only 15.6% of respondents in the north stated that a PDC should be conducted ‘every 6 months’. In order to establish why these recommendations differ throughout Germany, one would have to investigate the academic material taught at each university clinic. Another possibility for these differences could be the insufficient awareness of oral health in northern Germany. If this were true, more frequent checkups, individualised instructions and more frequent motivation would be required, in turn explaining the shorter interval of 3 months. This should also be investigated in another study on oral health in Germany. In general, the literature is in favour of a regular PDC. This is performed by a specialist and helps to maintain good oral hygiene throughout the treatment period [33, 34, 35, 36]. For fixed equipment, a PDC is recommended every 3–4 months [29].

### 4.3 Cleaning Techniques

A comparison of the recommendations for RA and MBA shows that Bass and Fones techniques were clearly the most popular brushing techniques. The increased occurrence of the simpler Fones technique in removable devices could be due to the younger age of patients with RA. The more complicated Bass technique was usually preferred for the ‘older’ patients with MBA since they have the manual skills to learn a technically more demanding cleaning technique.

There are very few articles and studies in literature on the level effectiveness of the different tooth brushing techniques, that all present very different results [38]. The recommendations of the surveyed orthodontists in the present study are more clear than the recommendations found in various literature sources: Poyato‐Ferrera et al. could prove that the Bass technique is superior to their own customary cleaning technique [36]. Harnacke et al. [39, 41] showed that the Fones technique is preferable to the Bass technique. The Fones technique displayed a more obvious plaque reduction. Sander et al. [29] recommend the Fones or Bass technique for patients with MBA. Arai et al. [59] proved that the Fones and scrubbing techniques are more effective than the modified Stillman, Roll and Bass technique. Gibson und Nassar et al. [34, 42] couldn't tell the difference between the Bass and Stillman techniques. In addition, both techniques are not sufficient for plaque reduction [42]. Zachrisson et al. [9] recommend horizontal techniques such as scrubbing and the Bass technique for MBA. Robinson und Nassar et al. [35, 43] couldn't tell the difference between the scrubbing and Bass techniques.

Despite a wide range of tooth brushing methods, most patients use their own personal technique, which is usually comparable to the scrubbing method [36, 38]. The recommendations of the German orthodontists in this study are valid, despite contradictory references. In most studies Fones and Bass technique are mentioned and tested [38]. Both are part of studies in which they are described as the method of choice [9, 36, 39, 41, 60]. Various dental associations recommend the modified Bass technique, the Fones technique and the scrubbing technique [38]. The Fones technique is also one of the best known techniques in Germany [61]. This could explain the clear decision the surveyed orthodontists have for these two tooth brushing methods.

The recommendation of a certain technique by the orthodontist and the application and implementation of the technique by the patient are equally important. In the study by Poyato‐Ferrera et al. [36], the participants succeeded in implementing the modified Bass technique, and the supragingival plaque was significantly reduced. However, it remains questionable whether the technology was still implemented outside of the study. Harnacke et al. [39] showed that the Fones technique is easier to learn than the Bass technique. The plaque values were also lower using the Fones technique than the Bass technique. In both groups, however, there were participants who did not keep up with the required technique until the end of the study. The reasons given were time pressure and convenience. This study shows that the simpler Fones technique is more successful than the more complicated Bass technique. This suggests that the Bass technique may be too difficult to learn or too cumbersome to use. The implementation of such brushing techniques seem to require a certain ambition and manual dexterity. Whether the motivation to apply a new cleaning technique is present in everyday life after completion of such a study also remains unknown. According to Sharma et al. [62], manual dexterity is age‐dependent. This study also shows that the recommendation and instruction of a certain cleaning method does not guarantee that it will be implemented at home.

Different tooth brushing methods alone, such as Bass and Roll methods, do not lead to the desired plaque reduction [42], therefore new ideas to improve plaque removal must be considered. One method to motivate the patient to brush thoroughly is to add bacterial plaque staining colourant to a toothpaste [63]. Until now, these plaque indicators have only been used in dental practices to demonstrate optimal oral hygiene to patients. However, plaque indicators are particularly advantageous for patients with MBA, as they are very susceptible to plaque accumulation [64]. In their 2016 study, Stevens et al. [63] compared a plaque‐indicator toothpaste with a comparable non‐indicator toothpaste. Participants who used toothpaste with indicator showed a plaque reduction of 51.3% (*p* = 0.015). The plaque reduction in patients who used normal toothpaste without an indicator was only 8.3% (*p* = 0.189). However, this study also demonstrated that the reduction of bacterial plaque is only so statistically significant if the participants receive additional instructions on the use of plaque indicator toothpaste.

This study is representative for Germany, because the data were collected here. Depending on factors like different marketing strategies of oral hygiene product producers, the findings might not apply to other countries.

## 5 Conclusion

The dental assistant instructs the patients orally. The MBA control is performed monthly and bimonthly for RA. PDC usually takes place every 6 months.

## 6 Clinical Relevance

### 6.1 Scientific Rationale for Study

Oral hygiene plans vary between orthodontists. This study analyses which oral hygiene instructions and treatments are given to the patient and by whom—the orthodontist or the dental hygienist—and which are most common in Germany and thus supported to be most effective.

### 6.2 Principal Findings

This study concludes that the dental assistant verbally instructs patients on their oral hygiene plan in most orthodontic practices in Germany. MBA patients are scheduled for appointments every month and RA patients are scheduled bimonthly. The Fones and Bass brushing techniques are widely favoured, and it is important to motivate patients to practice proper oral hygiene.

### 6.3 Practical Implications

Orthodontists should provide thorough oral health treatment plans and support during orthodontic treatment to prevent WSL and gingivitis. Professional dental cleaning, cleaning techniques and professional instruction are all key components of a successful orthodontic treatment.

## Author Contributions

C.B. conducted the research and recruited the sample; D.O. was data manager; C.E., D.O., P.F.P. and A.R.M. designed the research project and supervised the research; I.S., as statistician, analyzed and interpreted the data. C.B., I.S., D.O., C.E., P.F.P., A.R.M. and H.W. drafted and finalised the manuscript.

## Funding

No external funding was received for this study. This study was conducted at the University Medical Centre in Mainz and was supported by internal funds from the budget for research.

## Ethics Statement

This study's Ethics Approval Number is 11316. The study complies with the principles of the Declaration of Helsinki and the Declaration of Good Clinical Practice.

## Consent

The authors have nothing to report.

## Conflicts of Interest

The authors declare no conflicts of interest.

## Supporting information


**Data S1:** idh70028‐sup‐0001‐AppendixS1.docx.

**Data S2:** idh70028‐sup‐0002‐AppendixS2.pdf.

## Data Availability

All data generated or analysed during this study is included in this published article [and its Supporting Information files].
