# Supplementary material for: Oral Hygiene Recommendations From Orthodontists to Orthodontic Patients
Source: Int J Dent Hyg. 2026 Feb 11;24(2):237–46. doi: 10.1111/idh.70028 (PMC13050386; doi:10.1111/idh.70028)
Supplement: Supplementary file 2 — Data S2: idh70028‐sup‐0002‐AppendixS2.pdf. [file IDH-24-237-s001.pdf]

Anwendung von Mundhygieneinstruktionen und Bracketumfeldversiegelern/ Schutzlacken als Prophylaxemaßnahmen in der Kieferorthopädie

Fragebogen

Studienleitung:

OÄ Dr. Christina Erbe
Poliklinik für Kieferorthopädie, Klinik für Zahn-, Mund- und Kieferkrankheiten
Universitätsmedizin der Johannes Gutenberg-Universität Mainz
55131 Mainz

IMBE Institut für Medizinische Biometrie Epidemiologie und Informatik

Rückadresse
Institut für Medizinische Biometrie, Epidemiologie und Informatik
Universitätsmedizin der Johannes Gutenberg-Universität Mainz
Gebäude 902
Obere Zahlbacher Straße 69
55131 Mainz
Telefax: +49 (0) 6131 17-55 69

I. Allgemeine Angaben

1. Bitte geben Sie die Anzahl der kieferorthopädischen Behandler in Ihrer Praxis an:

________ Behandler

2. Seit wie vielen Jahren sind Sie als Kieferorthopäde/in tätig?

- ☐ <5 Jahren
- ☐ 5 – 10 Jahren
- ☐ 11 – 15 Jahren
- ☐ >25 Jahren

4. **Bitte geben Sie Ihre PLZ an:**

|__|__|__|__|__|

5. **Welcher Altersgruppe gehören Sie an:**

- ☐ 25 – 35 Jahre
- ☐ 36 – 45 Jahre
- ☐ 46 – 55 Jahre
- ☐ 56 – 65 Jahre
- ☐ 66 – 75 Jahre

II. Mundhygiene Anweisung

1. 8.1.1.1 Wer führt in Ihrer Praxis die **Mundhygieneanweisungen** durch?

☐ Behandler ☐ ZFA

2. Welche **Mundhygiene-Produkte** empfehlen Sie bei Patienten mit festsitzenden kieferorthopädischen Apparaturen? _(Mehrfachantworten möglich)_

**Vestibuläre** Multibracketapparaturen		**Linguale** Multibracketapparaturen	
• Zahnbürste	☐ manuell	• Zahnbürste	☐ manuell
	☐ elektrisch		☐ elektrisch
	☐ Schall		☐ Schall
	☐ Interdentalbürstchen		☐ Interdentalbürstchen
•	☐ Gelée	•	☐ Gelée
Flouridhaltiges	☐ Mundspüllösung	Flouridhaltige	☐ Mundspüllösung

3. Welche **Mundhygiene-Produkte** empfehlen Sie bei Patienten mit **herausnehmbaren** kieferorthopädischen Apparaturen? _(Mehrfachantworten möglich)_

• Zahnbürste
- ☐ manuell
- ☐ elektrisch
- ☐ Schall

	☐ Interdentalbürstchen
•	☐ Gelée
Flouridhaltiges	☐ Mundspüllösung

4. **Wie werden Ihre Patienten bzgl. der Mundhygiene instruiert?**

☐ mündliche Aufklärung ☐ Infobroschüre ☐ Putzschulung ☐ anderes

5. **Wie oft bestellen Sie Ihre Patienten mit festsitzenden Apparaturen ein?**

☐ monatlich ☐ 2-monatlich ☐ 3-monatlich

6. **Wie oft bestellen Sie Ihre Patienten mit herausnehmbaren Apparaturen ein?**

☐ monatlich ☐ 2-monatlich ☐ 3-monatlich

III. Mundhygiene Kontrolle

1. **Wie oft kontrollieren Sie (Behandler) die Mundhygiene Ihrer Patienten?**

☐ bei jeder Sitzung ☐ alle |___|___| Monate

2. **In welchem Abstand führen Sie eine professionelle Zahnreinigung (PZR) durch?**

☐ alle |___|___| Monate

3. **Führen Sie in Ihrer Praxis regelmäßige PZR durch?**

☐ ja ☐ ja, unregelmäßig ☐ nein

4. Wer führt die PZR bei Ihnen in der Praxis durch?
☐ selbst (Behandler) ☐ ZFA ☐ Überweisung Zahnarzt

5. Welche Putztechnik empfehlen Sie bei kieferorthopädischen Apparaturen?

**Herausnehmbare** Apparaturen	**Multibracket**-Apparaturen
☐ alle Zahnflächen kreisend (und von Rot nach Weiß)	☐ alle Zahnflächen kreisend (und von Rot nach Weiß)
☐ unter 45° zur Gingiva, Außen- und Innenflächen, dann von Rot nach Weiß	☐ unter 45° zur Gingiva, Außen- und Innenflächen, dann von Rot nach Weiß
☐ von Rot nach Weiß	☐ von Rot nach Weiß
☐ kreisend über die Gingiva zum Zahn	☐ kreisend über die Gingiva zum Zahn
☐ Schrubb-Technik, parallel zu den Zahnreihen	☐ Schrubb-Technik, parallel zu den Zahnreihen

IV. Anwendung und Kontrolle des Bracketumfeldversiegler

1. **Verwenden Sie Bracketumfeldversiegler/Schutzlacke als Prophylaxemaßnahme**?
☐ ja ☐ nein

2. Welche Produkte kommen bei Ihnen zum Einsatz und wie viele Auftragungen erfolgen bei <u>einer</u> Applikation?

**Produkte**	**Anzahl an Auftragungen bei <u>einer</u> Sitzung**
☐ Fluor Protector (Ivoclar Vivadent)	☐ 1x ☐ 2x ☐ 3x
☐ Cervitec Plus (Ivoclar Vivadent)	☐ 1x ☐ 2x ☐ 3x

☐	Protecto CaF2 Nano (Bonadent)	☐ 1x	☐ 2x	☐ 3x
☐	Protecto F (Bonadent)	☐ 1x	☐ 2x	☐ 3x
☐	Maximum Cure (Reliance Orthodontic)	☐ 1x	☐ 2x	☐ 3x
☐	Light Bond (Reliance Orthodontic Products)	☐ 1x	☐ 2x	☐ 3x
☐	Clinpro XT Varnish (3M ESPE)	☐ 1x	☐ 2x	☐ 3x
☐	Alpha-Glaze (Rocky Mountain Orthodontics)	☐ 1x	☐ 2x	☐ 3x
☐	ProSeal (Reliance Orthodontics)	☐ 1x	☐ 2x	☐ 3x
☐	Ortho Solo (Ormco)	☐ 1x	☐ 2x	☐ 3x
☐	andere: ____________________	☐ 1x	☐ 2x	☐ 3x

3. Kontrollieren Sie die Versiegelung auf Beschädigungen?

☐ Nein, nicht notwendig ☐ Ja, bei den Sitzungen

4. Wie bzw. mit welcher Methode kontrollieren Sie des Versiegelungserfolg des angewendeten Produktes? (Mehrfachantworten möglich)

visuell: ☐ auf deckende Wirkung Taktil: ☐ Sonde

☐ auf Reflexionsveränderung zur Umgebung

5. Stellen Sie bei Ihren Patienten nach Multibracketbehandlungen sichtbare Demineralisationen (Weißverfärbungen) fest?

☐ häufig ☐ manchmal ☐ selten

V. Kommentare und Bemerkungen

1. **Haben Sie noch weitere Kommentare zu diesem Fragebogen?**

____

____

2. Möchten Sie über die Ergebnisse dieser Studie informiert werden?

☐ ja ☐ nein

Vielen Dank für die Beantwortung der Fragen!
